# Expected‐value bias in routine third‐trimester growth scans

**DOI:** 10.1002/uog.21929

**Published:** 2020-03-02

**Authors:** L. Drukker, R. Droste, P. Chatelain, J. A. Noble, A. T. Papageorghiou

**Affiliations:** ^1^ Nuffield Department of Women's & Reproductive Health University of Oxford Oxford UK; ^2^ Institute of Biomedical Engineering University of Oxford Oxford UK

**Keywords:** artificial intelligence, ascertainment bias, detection bias, expectancy bias, eye tracking, fetal biometry, growth scan, observer bias, observer effect, ultrasound

## Abstract

**Objectives:**

Operators performing fetal growth scans are usually aware of the gestational age of the pregnancy, which may lead to expected‐value bias when performing biometric measurements. We aimed to evaluate the incidence of expected‐value bias in routine fetal growth scans and assess its impact on standard biometric measurements.

**Methods:**

We collected prospectively full‐length video recordings of routine ultrasound growth scans coupled with operator eye tracking. Expected value was defined as the gestational age at the time of the scan, based on the estimated due date that was established at the dating scan. Expected‐value bias was defined as occurring when the operator looked at the measurement box on the screen during the process of caliper adjustment before saving a measurement. We studied the three standard biometric planes on which measurements of head circumference (HC), abdominal circumference (AC) and femur length (FL) are obtained. We evaluated the incidence of expected‐value bias and quantified the impact of biased measurements.

**Results:**

We analyzed 272 third‐trimester growth scans, performed by 16 operators, during which a total of 1409 measurements (354 HC, 703 AC and 352 FL; including repeat measurements) were obtained. Expected‐value bias occurred in 91.4% of the saved standard biometric plane measurements (85.0% for HC, 92.9% for AC and 94.9% for FL). The operators were more likely to adjust the measurements towards the expected value than away from it (47.7% *vs* 19.7% of measurements; *P* < 0.001). On average, measurements were corrected by 2.3 ± 5.6, 2.4 ± 10.4 and 3.2 ± 10.4 days of gestation towards the expected gestational age for the HC, AC, and FL measurements, respectively. Additionally, we noted a statistically significant reduction in measurement variance once the operator was biased (*P* = 0.026). Comparing the lowest and highest possible estimated fetal weight (using the smallest and largest biased HC, AC and FL measurements), we noted that the discordance, in percentage terms, was 10.1% ± 6.5%, and that in 17% (95% CI, 12–21%) of the scans, the fetus could be considered as small‐for‐gestational age or appropriate‐for‐gestational age if using the smallest or largest possible measurements, respectively. Similarly, in 13% (95% CI, 9–16%) of scans, the fetus could be considered as large‐for‐gestational age or appropriate‐for‐gestational age if using the largest or smallest possible measurements, respectively.

**Conclusions:**

During routine third‐trimester growth scans, expected‐value bias frequently occurs and significantly changes standard biometric measurements obtained. © 2019 the Authors. *Ultrasound in Obstetrics & Gynecology* published by John Wiley & Sons Ltd on behalf of the International Society of Ultrasound in Obstetrics and Gynecology.

**CONTRIBUTION**

*What are the novel findings of this work?*

A detailed, novel analysis of full‐length routine fetal growth scans and sonographer eye tracking has shown that operators are at risk of expected‐value bias when acquiring fetal biometry measurements.

*What are the clinical implications of this work?*

When making clinical decisions, clinicians should be aware that estimated fetal weight may be inaccurate due to expected‐value bias. Ultrasound operators should be aware of this potential bias while performing biometric measurements.

## INTRODUCTION

In science, the accuracy of measurement is a crucial prerequisite for correct interpretation of results. There are many reasons for inaccurate measurement and one that is relatively easy to overcome is the observer bias, which is the tendency to see what we expect to see^1^. The observer bias is also known as expected‐value bias, detection bias, observer–expectancy effect, expectancy bias, observer effect or ascertainment bias. This bias may occur if the observer has a preconceived idea of what a measurement ought to be, leading to adjustments of the readings. Hróbjartsson and colleagues^2^ undertook a systematic review quantifying the impact of observer bias, by comparing estimates between studies in which outcome assessors were blinded to the intervention and those in which outcome assessors were not blinded. For clinical trials that used measurement scale outcomes, non‐blinded outcome assessment exaggerated the effect size by as much as 68%^2^. In randomized trials, blinding is used to reduce bias and usually involves preventing knowledge of which intervention or control is being received by a study participant^3^, ^4^. Day and Altman^5^ highlight that blinding is important in other types of research too, such as evaluation of the performance of a diagnostic test and reproducibility of measurement techniques. Blinding makes it difficult to bias results intentionally or unintentionally and so helps to ensure the credibility of measurements^5^. Recently, a review of systematic error and cognitive bias in obstetric ultrasound suggested that expectation bias is pertinent to obstetric ultrasound studies^6^.

**Figure 1:**
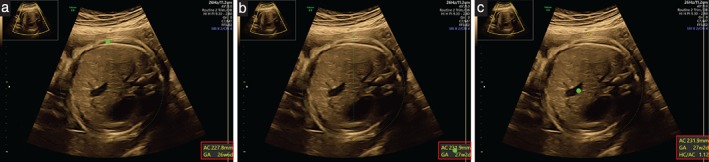
Occurrence of expected‐value bias during measurement of fetal abdominal circumference at 28 + 0 weeks' gestation. Red rectangle outlines measurement box and green dot has been added to represent operator eye focus (not visible to operator during scan). (a) Caliper adjustment in progress. (b) Operator eye fixation on measurement box detected, suggesting biased measurement. (c) Measurement accepted.

In contrast to trials, measurement blinding is not usually carried out in day‐to‐day clinical management. This may be of particular relevance in fetal growth assessment, which looks for aberrations from normally expected growth patterns; however, blinding of the examiner to the gestational age of the pregnancy to avoid the effect of clinician bias is rarely practiced. During clinical assessment of fundal height, the guidance suggests that caregivers should hold the tape in a way that the measurement cannot be seen^7^. This is however not usually the case in ultrasound assessment; during a routine growth scan, comprising the three standard biometric plane measurements of head circumference (HC), abdominal circumference (AC) and femur length (FL)^8^, the ultrasound machine will usually display the reading value (circumference or length, in mm or cm) as well as an observed gestational age (in weeks + days) corresponding to the measurement (Figure 1). This can lead to an observer (or expected‐value) bias, which means that the operator may adjust the circumference or length so that the observed gestational age matches the gestational age calculated previously by dating. In turn, this may lead to a biased fetal growth estimation. The use of blinding in this scenario would overcome such bias. Although blinding of the operator to the actual gestational age or to the machine‐displayed values during growth scan assessment has been done in some studies^9^, ^10^, measurement blinding is rarely used in routine clinical practice^11^.

In this study we aimed to evaluate the incidence of expected‐value bias in routine fetal growth scans and assess its impact on standard biometric measurements.

## METHODS

This was a prospective study of routine ultrasound scans performed between May 2018 and August 2019 in women with a singleton pregnancy, by sonographers and fetal medicine doctors at the Maternity Ultrasound Unit, Oxford University Hospitals National Health Service (NHS) Foundation Trust, Oxfordshire, UK. In this center, all women are offered three routine ultrasound scans: first‐trimester crown–rump length dating^12^ at approximately 12 weeks' gestation, which includes nuchal translucency measurement for first‐trimester aneuploidy screening; a 20‐week anomaly scan; and a 36‐week growth scan in which estimated fetal weight (EFW) is computed^13^. Additionally, based on risk factors or clinical indications, women may be offered additional scans at other gestational ages^14^. Ultrasound examinations are carried out or supervised by accredited sonographers or fetal medicine doctors using standard ultrasound equipment. For quality control measures, the stored images and the reliability of measurements are regularly assessed using the INTERGROWTH‐21^st^ quality control criteria^15^. Inclusion criteria were maternal age > 18 years of age and the ability to provide verbal and written informed consent in English.

This study was part of a project entitled Perception Ultrasound by Learning Sonographic Experience (PULSE)^16^. This is an innovative interdisciplinary project designed to apply the latest ideas from machine learning and computer vision to build, from real‐world video data and other sensory data, computational models that describe how an expert sonographer performs a diagnostic study of a subject from multiple perceptual cues. By understanding closely how experts learn and undertake diagnostic ultrasound, we believe that we can build considerably more powerful assistive interpretation methods than have been possible so far. As part of the PULSE project, full‐length routine ultrasound scan videos are captured and recorded, probe movement is recorded and the point‐of‐gaze of the sonographer on the monitor of the ultrasound scanner is tracked.

All ultrasound scans included in this study were performed using commercial Voluson E8 version BT18 (GE Healthcare, Zipf, Austria) ultrasound machines, equipped with standard curvilinear (C2‐9‐D, C1‐5‐D) and three‐dimensional/four‐dimensional (RAB6‐D) probes. Synchronized eye tracking was undertaken using an eye tracker (Tobii Eye‐tracking Eye Tracker 4C, Danderyd, Sweden) attached to the ultrasound machine; the validity of eye‐tracking has been reported previously^17^. Of note, only one of the ultrasound machines in the center is equipped with eye‐tracking and recording devices, which limited the number of patients recruited during the study period.

This study was approved by the UK Research Ethics Committee (Reference 18/WS/0051) and written informed consent was given by all participating pregnant women. Sonographers also consented to participate in the study at the outset but did not have any visual or other signal to know that the tracking devices were functioning during the examination.

Funding for this study was granted by the European Research Council (ERC‐ADG‐2015 694 581, project PULSE) and the Engineering and Physical Sciences Research Council (EPSRC EP/M013774/1, project Seebibyte).

### Biometric measurement acquisition

Acquisition of the three standard biometric measurements (i.e. HC, AC and FL) is a three‐stage process. First, the operator obtains an optimal acquisition of a standard biometric plane and freezes it on the screen. Next, the operator measures the biometric variable by placing calipers on the image; automatic caliper placement is turned on by default on the ultrasound machines used in our unit. The operator will often adjust caliper placement to achieve the best visual fit. During caliper placement and adjustment, ultrasound machines display on the screen a measurement box in which the measured length or circumference (in cm or mm) and the gestational age corresponding to the measurement (in weeks + days) are shown and updated in real‐time (Figure 1). Finally, the operator accepts the standard biometric plane measurement by saving the image with a visible measurement.

### Data extraction

Each scan was automatically analyzed on a video frame‐by‐frame basis using a purpose‐built software program implemented in Python (http://www.python.org, version 3.7.0) using OpenCV (http://www.opencv.org, version 3.4) and Tesseract (http://www.github.com/tesseract-ocr, version 3.05). For each scan videoclip, the software program first detected the episodes of measuring a standard biometric plane by the appearance of the measurement box. Next, for each standard biometric measurement, the program detected uninterrupted fixations of the operator's eye on the measurement box lasting ≥ 100 ms, which is a widely accepted lower limit for eye fixation^18^. If eye fixation was interrupted, it was considered as one single episode of eye fixation if this interruption lasted ≤ 400 ms, or as a separate eye‐fixation episode if it lasted > 400 ms^18^, ^19^. Additionally, we verified the threshold for eye fixation by randomly looking at more than 50 detected fixations and ensuring that the threshold resulted in no false positives.

Concurrently, the software program stored the values displayed in the measurement box when the calipers were initially placed and when the operator accepted the measurement. Additionally, the software program stored the values displayed in the measurement box upon each detection of eye fixation on the measurement box. The measurement box values and parameters were extracted via optical character recognition.

The Voluson E8 BT18 machine, by design, displays the observed (measured) gestational age as ‘OOR’ (out of range) in the measurement box when no standard curve is available for the measurement or the available curve does not cover the extremes of gestational age. In the current analysis, when this happened, the gestational‐age values were computed using the appropriate original formula.

Expected value was defined as the gestational age at the time of the fetal growth scan, calculated based on the estimated due date that was established at the dating scan. Observed value was defined as the gestational age displayed in the measurement box which was based on the standard biometric measurement.

Expected‐value bias was defined as occurring when the operator looked at the measurement box during the process of caliper adjustment before saving a standard biometric measurement (Figure 1 and Videoclip S1).

After a specific standard biometric measurement (either HC, AC or FL) was saved, any additional same standard biometric measurement saved during the same examination was considered a repeat measurement.

To evaluate the incidence of expected‐value bias we evaluated whether the operator looked at the measurement box before saving a standard biometric plane. To assess the impact of expected‐value bias, we: (I) measured how often the operators adjusted the calipers toward or away from the expected value; (II) evaluated the deviation of the observed from the expected values before and after the expected‐value bias took place (i.e. at the time the operator looked at the measurement box for the first time and when the measurement was saved); (III) compared the deviation between the observed and expected gestational age for standard biometric measurements that were repeated *vs* those that were not; and (IV) evaluated the impact of expected‐value bias on the EFW by calculating the lowest and highest possible EFW using the smallest and largest HC, AC, and FL measurements, respectively, before and after expected‐value bias occurred.

### Statistical analysis

We report descriptive statistics. Continuous variables were compared using the Student's *t*‐test, Wilcoxon signed‐rank test (paired) or Mann–Whitney *U*‐test (unpaired). Comparison between saved (accepted) measurements and those recorded when the operator looked for the first time at the measurement box was investigated using multiple linear regression models. In order to evaluate independent relationships between the number of repeat measurements and the absolute deviation from the actual gestational age (expected value), we conducted a multifactor ANOVA analysis. Analyses were adjusted for the body mass index (BMI) of the pregnant woman and the number of years' scanning experience of the operator. *P*‐values < 0.05 were considered statistically significant. Analyses were carried out using R (http://www.r-project.org, version 3.5.2), Python (http://www.python.org, version 3.7.0), Pandas (http://pandas.pydata.org, version 0.24.0), SciPy (http://www.scipy.org, version 1.1.0) and Matplotlib (http://www.matplotlib.org, version 3.0.0).

## RESULTS

During the study period, a total of 272 women undergoing a routine third‐trimester fetal growth scan were recruited. Demographic characteristics of the participants are displayed in Table 1. The mean gestational age at the time of the fetal growth scan was 34.6 ± 3.1 weeks. The examinations were performed by 16 operators, of which nine were accredited sonographers and seven fetal medicine doctors, with a median of 3 years' (range, 4 months to 14 years) clinical post‐accreditation experience in sonography (Table 2).

**Table 1:** Characteristics of 272 women with singleton pregnancy included in study cohort

Characteristic	Value
Maternal age (years)	31.9 ± 5.7
Smoker at booking	21 (7.7)
BMI at < 15 weeks (kg/m^2^)	25.8 ± 5.3
Conception by IVF	4 (1.5)
Nulliparous	123 (45.2)
GA at fetal growth scan (weeks)*	34.6 ± 3.1
Pregnancy dating by CRL	249 (91.5)
Pre‐eclampsia	7 (2.6)
Gestational diabetes mellitus	11 (4.0)
Preterm birth	11 (4.0)
Vaginal birth	203 (74.6)

Data are given as mean ± SD or *n* (%).

* Gestational age (GA) based on estimated due date established at dating scan.

BMI, body mass index; CRL, crown–rump length; IVF, *in‐vitro* fertilization.

**Table 2:** Characteristics of 16 ultrasound operators who participated in study

Characteristic	Value
Gender	
Female	14 (87.5)
Male	2 (12.5)
Clinical experience in scanning	
< 2 years	3 (18.8)
2–5 years	7 (43.8)
5–10 years	5 (31.3)
> 10 years	1 (6.3)
Accreditation	
Sonographer	9 (56.3)
Fetal medicine doctor	7 (43.8)

Data are given as *n* (%).

A total of 1409 standard biometric plane measurements were made in the 272 scans, comprising 354 of the HC, 703 of the AC and 352 of the FL. We observed a risk of measurement bias in 91.4% of the measurements, of which 85.0%, 92.9% and 94.9% were of the HC, AC, and FL measurements, respectively (Table 3). Importantly, there was evidence that looking at the measurement box during caliper adjustment was likely due to bias rather than due to other reasons, as operators were more likely to adjust measurements towards the expected gestational age than to adjust it away from the expected gestational age (47.7% *vs* 19.7% overall; 49.5% *vs* 16.4% for HC; 51.5% *vs* 26.3% for AC; and 38.9% *vs* 9.6% for FL; *P* < 0.001 for all comparisons) (Table 3).

**Table 3:** Number of measurements performed during fetal growth scan and incidence of expected‐value bias, according to standard biometric measurement

Standard biometric measurement	Saved measurements(*n*)	Repeat measurements(*n*)	Measurements per growth scan (mean ± SD)	Biased measurements(%)	Adjustment of measurement			
					Towards expected GA* (%)	Away from expected GA* (%)	Mean adjustment towards expected GA* (days' gestation)	*P*
Head circumference	354	82	1.3 ± 0.6	85.0	49.5	16.4	2.3 ± 5.6	< 0.001
Abdominal circumference	703	431	2.6 ± 1.0	92.9	51.5	26.3	2.4 ± 10.4	< 0.001
Femur length	352	80	1.3 ± 0.7	94.9	38.9	9.6	3.2 ± 10.4	< 0.001
Total	1409	593	5.2 ± 1.7	91.4	47.7	19.7	2.6 ± 9.5	< 0.001

* Gestational age (GA) based on estimated due date established at dating scan.

The risk of expected‐value bias applied to all operators, though it varied from 56% to 100% of measurements for the different operators. The correlation between years of scanning experience of an operator and the percent of measurements prone to bias was not statistically significant (*P* = 0.34).

**Figure 2:**
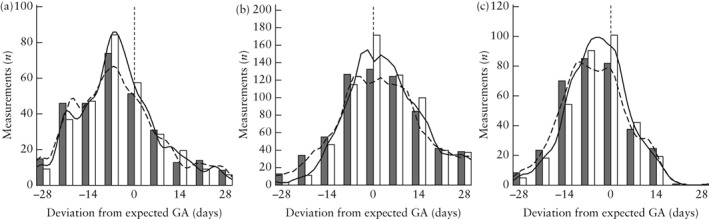
Deviation of observed gestational age (GA), based on standard biometric measurement of head circumference (a), abdominal circumference (b) and femur length (c) at fetal growth scan, from expected GA, based on estimated due date established at dating scan, before (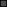, 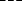) and after (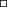, 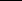) expected‐value bias occurred, i.e. when operator first looked at measurement box and after measurement was saved.

The deviation of the observed gestational age (based on the biometric measurement) from the expected gestational age, expressed in days of gestation, before and after expected‐value bias occurred, is presented in Figure 2. We found a statistically significant difference in the mean observed gestational age before and after the operators looked at the measurement box, with the HC, AC and FL measurements being closer to the expected gestational age by 2.3 ± 5.6, 2.4 ± 10.4 and 3.2 ± 10.4 days of gestation, respectively (*P* < 0.001 for all comparisons). Additionally, we noted that values were closer to the mean after measurement bias occurred (reduction of the variance, Levene's test, *P* = 0.0255). These correlations remained statistically significant after multivariable analysis was performed, adjusting for maternal BMI and years' scanning experience of the operator as confounding variables. Additionally, when there was evidence of bias, we compared the measurement at the time the operator first looked at the measurement box and that eventually saved. We noted that the further the initial measurement was from the expected value, the larger was the adjustment of calipers toward the expected value (*P* < 0.001 for AC, HC and FL). This correlation remained significant after adjusting for operator experience and maternal BMI.

**Figure 3:**
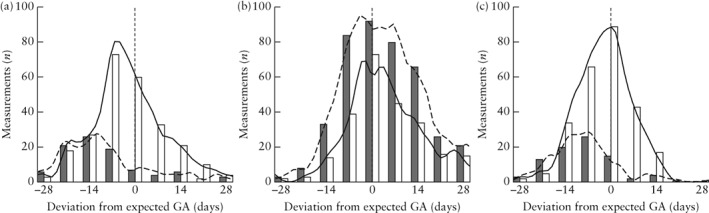
Deviation of observed gestational age (GA), based on standard biometric measurement of head circumference (a), abdominal circumference (b) and femur length (c) at fetal growth scan, from expected GA, based on estimated due date established at dating scan, for measurements that were repeated (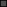, 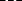) and those that were not repeated (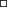, 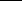).

We also compared the deviation of the observed from the expected gestational age between measurements that were repeated and those that were not. A total of 82, 431 and 80 measurements of HC, AC and FL, respectively, were repeated. Operators were more likely to repeat a measurement when this was far from the expected value. The observed gestational age was significantly closer to the expected gestational age for measurements that were not repeated. This means that the operators were more likely to acquire another image of the same standard biometric plane and measure again if the initial measurement was far from the expected value. The mean deviation of the observed from the expected gestational age for measurements that were repeated, compared with those that were not, was 15.1 ± 8.4 *vs* 10.2 ± 10.9 gestational days (*P* < 0.001) for HC measurements; 12.4 ± 14.3 *vs* 11.5 ± 12.3 days (*P* = 0.036) for AC measurements; and 13.3 ± 11.1 *vs* 7.7 ± 9.7 days (*P* < 0.001) for FL measurements (Figure 3). This correlation remained statistically significant after performing multivariable analysis, adjusting for maternal BMI and operator experience.

Finally, in order to estimate the impact of this potential expected‐value bias, we calculated the lowest and highest EFW, using respectively the smallest and largest biased HC, AC and FL measurements. The discordance, expressed in percentage terms, was 10.1% ± 6.5%. The *Z*‐score difference between the highest and lowest possible EFW was 0.83 ± 0.58. This means that 46 fetuses (17%; 95% CI, 12–21%) could be considered as small‐for‐gestational age, if using the smallest possible measurements, and appropriate‐for‐gestational age, if using the largest possible measurements. Similarly, in 34 scans (13%; 95% CI, 9–16%) the fetus could be considered as large‐for‐gestational age or appropriate‐for‐gestational age if the largest or smallest possible measurements, respectively, were used.

## DISCUSSION

This study has demonstrated that measurements undertaken during fetal growth scans are often biased by knowledge of the gestational age and the expected measurement for gestation. Operators tend to correct caliper placement at the time of the scan toward the expected measurement for the actual gestational age. The amount of correction correlates with the amount of deviation from the expected value. Additionally, we noted that operators were more likely to retake an image and repeat a measurement when the first measurement was far from the expected value. We did not find a correlation between the tendency to undertake such correction and the number of years' clinical experience or type of accreditation of the operator.

It is difficult to compare our findings with previous reports, as observer bias/expected‐value bias is not well studied in obstetric ultrasound. Nevertheless, unbiased and accurate measurement is a fundamental tenet of science. Such bias is not limited only to obstetric ultrasound, but can be encountered in many other medical fields, and is known to modify significantly clinical measurements as well as experimental results^1^. For example, in the case of blood pressure measurement, having an expectation of what it ought to be, might lead to an arbitrary adjustment of a non‐automatic reading^20^.

The magnitude of the effect of expected‐value bias is difficult to ascertain and requires a study comparing blinded and non‐blinded fetal biometric measurements. Nevertheless, we found that the impact of bias on EFW may be as high as 10%, and that in 17% of scans the fetus could be considered as small‐for‐gestational age or appropriate‐for‐gestational age, depending on whether the smallest or the largest possible bias measurement was used. The corresponding figure for fetuses that could be considered as large‐for‐gestational age or appropriate‐for‐gestational age was 13%. This could lead to erroneous diagnosis of growth restriction, and thus to unnecessary intervention, maternal anxiety and iatrogenic perinatal morbidity, or it could result in classifying as normal a small‐for‐gestational‐age fetus, putting the pregnancy at risk for adverse perinatal outcome^21^. Hence, when making an obstetric decision, the possibility of bias in the estimation of fetal weight should also be taken into account. Moreover, in clinical practice, it is known that the detection rates for growth restriction during screening remain limited and one could hypothesize that expected‐value bias could be one of the reasons.

Our findings also have obvious and important implications on research that is based on routine clinical data acquisition, for example when studying normal fetal growth. Bias in measurements means that any underlying formula programmed into the ultrasound system, relating gestational age to the fetal measurement, will have an important effect when aggregating data. It is for this reason that blinding operators to the measurement value is such a crucial step when creating normal ranges^9^, ^10^, ^11^. In addition, this study is part of the PULSE project, which is designed to apply the latest ideas from artificial intelligence, machine learning and computer vision to build computational models that describe how expert sonographers perform scanning. Our findings emphasize the importance of minimizing bias when training computer models to perform a task. This is because artificial intelligence is trained by humans who may introduce their own biases to the learning process, resulting in biased models. Based on current practice, algorithm training to measure standard biometric planes might result in a built‐in bias when automatically calculating fetal biometry. This bias can potentially even be amplified by the algorithm^22^, ^23^.

In our study all fetal growth scans were routine assessments and most fetuses were appropriate‐for‐gestational age. It is possible that this bias may be more pronounced in pregnancies with small‐ and large‐for‐gestational‐age fetuses, as greater measurement correction towards the expected value would be anticipated. This may be compounded by the well‐documented larger errors in fetal weight estimation in small‐ and large‐for‐gestational‐age fetuses^24^.

The accuracy and reliability of fetal biometry measurements are determined by the accuracy of standardized biometric plane acquisition^25^ and caliper placement. In this study, to evaluate the effect of bias during caliper placement, we tracked the eye movements of the operator, considering that risk of bias occurred when the operator looked at the measurement box while adjusting caliper placement or saving the image. However, a biased measurement does not necessarily mean that the measurement is incorrect. Extreme values are likely to represent a low‐quality acquisition rather than a fetal growth concern. Therefore, operators may commonly look at the displayed measurement to ensure that their measurement meets their expectation before adjusting the calipers. Likewise, adjusting the measurement away from the actual gestational age does not necessarily represent an unbiased measurement. For example, if the operator is aware of gestational diabetes, the operator may unconsciously perceive that the fetus is big, and hence measure it to be large‐for‐gestational age. Nevertheless, our findings suggest that, on average, operators adjust the measurement towards the expected measurement for gestational age. Similarly, performing a repeat standard biometric plane acquisition and measurement may represent good practice^8^. Nonetheless, operators may choose to acquire an additional standard biometric measurement due to an unsatisfactory self‐scoring quality assurance^8^ or because of a measurement value that does not match closely enough the expected one. We noticed measurements that were not repeated were closer to the expected value.

Our study has some limitations. It was conducted in a single maternity unit which may not represent practice at other centers; nevertheless, we included 16 operators and the same finding was seen in all, making external validity more likely. In addition, even though the operators were aware that the scans and their eye movements were being recorded, they had not been informed of the aim of the current analysis meaning that it is unlikely that they acted differently while participating in this study. Another limitation is that the impact of expected‐value bias could only be estimated. To examine accurately the impact of bias would require performing a study in which operators are assigned randomly to blinding of measurements. However, the principle shown in this paper suggests that expected‐value bias is both common and clinically significant. We reported recently that operators rarely look at the safety indices while they scan^26^. This suggests that eye tracking of the operator is precise in detecting the point of gaze. The finding that operators look at measurements, but not bioeffects, is in accordance with our assumption. Finally, we used the actual gestational age as the reference (expected) value, however, in our setting this is based on a measurement performed at the dating scan, which may also be biased^27^.

In conclusion, observer bias towards expected values of fetal measurements is prevalent in routine third‐trimester growth scans. Further research should evaluate the added value of eliminating this bias to the overall accuracy of growth scans. To overcome it, ultrasound manufacturers should consider including settings that allow operators to be blinded before saving or ending ultrasound examinations.

## Supporting information


**Videoclip S1** Occurrence of expected‐value bias during measurement of abdominal circumference at 36 + 5 weeks' gestation. Initial observed (i.e. automatically calculated) gestational age is 38 + 6 weeks. After caliper adjustment, operator looks at measurement box that displays gestational age of 38 + 0 weeks. Then, operator adjusts caliper and looks again at measurement box. Final saved measurement equals gestational age of 37 + 2 weeks. Note that, in order to facilitate understanding of expected‐value bias, eye tracking is indicated on video by green dot; however, operator did not see this or any other indication of eye‐tracking function on screen during measurement.

**Videoclip S2** Presentation of study at 29^th^ ISUOG World Congress on Ultrasound in Obstetrics and Gynecology.
